# Facilitating identification of minimal protein binding domains by cross-linking mass spectrometry

**DOI:** 10.1038/s41598-017-13663-y

**Published:** 2017-10-18

**Authors:** Qingyang Liu, Sanne Remmelzwaal, Albert J. R. Heck, Anna Akhmanova, Fan Liu

**Affiliations:** 1 Cell Biology, Faculty of Science, Utrecht University, Padualaan 8, 3584 CH Utrecht, The Netherlands; 2 Biomolecular Mass Spectrometry and Proteomics, Bijvoet Center for Biomolecular Research and Utrecht Institute for Pharmaceutical Sciences, Utrecht University, 3584 CH Utrecht, The Netherlands; 3 Leibniz Institute of Molecular Pharmacology (FMP), Robert-Rössle-Straße 10, 13125 Berlin, Germany

## Abstract

Characterization of protein interaction domains is crucial for understanding protein functions. Here we combine cross-linking mass spectrometry (XL-MS) with deletion analysis to accurately locate minimal protein interaction domains. As a proof of concept, we investigated in detail the binding interfaces of two protein assemblies: the complex formed by MICAL3, ELKS and Rab8A, which is involved in exocytosis, and the complex of SLAIN2, CLASP2 and ch-TOG, which controls microtubule dynamics. We found that XL-MS provides valuable information to efficiently guide the design of protein fragments that are essential for protein interaction. However, we also observed a number of cross-links between polypeptide regions that were dispensable for complex formation, especially among intrinsically disordered sequences. Collectively, our results indicate that XL-MS, which renders distance restrains of linked residue pairs, accelerates the characterization of protein binding regions in combination with other biochemical approaches.

## Introduction

Proteins are the primary effectors of the cell. They execute a plethora of cellular processes, which depend on protein-protein interactions (PPIs) responsible for formation of stable protein complexes and dynamic interaction networks^1,2^. Many PPIs are mediated by protein domains. These structural units are defined by their topological conformations, forming stable and compact three-dimensional organizations independent of other regions of the protein^3,4^. Additionally, PPIs can also involve linear motifs located in intrinsically unstructured protein regions^5–7^. Characterization of protein segments directly involved in PPIs allows their targeted disruption, which is crucial for developing a molecular-level understanding of various cellular processes.

For decades, numerous techniques have been introduced to determine protein interaction domains, among which affinity purification combined with deletion analysis (generation of deletion mutants) is one the most popular methods^8,9^. In this approach, a specific gene of interest is cloned into a suitable host for (over)expression of the desired protein segment. Subsequently, affinity purification-based assays such as immunoprecipitations or pull-downs are performed to investigate the interaction between the (over)expressed bait protein and its binding partners. However, generating serial truncated proteins can be time-consuming and labour-intensive, especially when the protein of interest is large and contains many domains^10^. Furthermore, designing appropriate protein fragments may also be a challenge, as the current approaches, such as predictions from protein structural information in the Protein Data Bank (PDB) or through computational algorithms, often do not render precise results^4,11^.

Chemical cross-linking mass spectrometry (XL-MS) is another valuable technique to characterize protein interactions as well as their structural basis at the peptide level^12,13^. In XL-MS experiments, cross-linking reagents, typically containing two reactive groups linked by a space arm, covalently connect two amino acid residues that are close in space. Subsequently, mass spectrometry is used to identify the cross-link and its corresponding linked residues. These experiments provide distance proximities (i.e., the maximum distance restraint between the two linked residues), which can facilitate the characterization of protein interactions as well as their binding interfaces^14–18^. Although XL-MS does not provide atomic structural information, which may be a shortcoming comparing to other high-resolution methods, such as X-ray crystallography and cryo-EM, it remains a very attractive approach because of several other advantages. For instance, XL-MS experiments only require a modest amount and purity of proteins, hence readily applicable to various endogenous proteins or protein complexes directly after a small-scale affinity purification. Another advantage of the XL-MS approach is the convenience and promptness, as the experiments can be accomplished in several days. Third, the performance XL-MS is independent of the length and the tertiary structure of proteins, and is therefore beneficial in cases that are challenging for other methods, such as analyses of large proteins and disordered regions.

**Figure 1:**
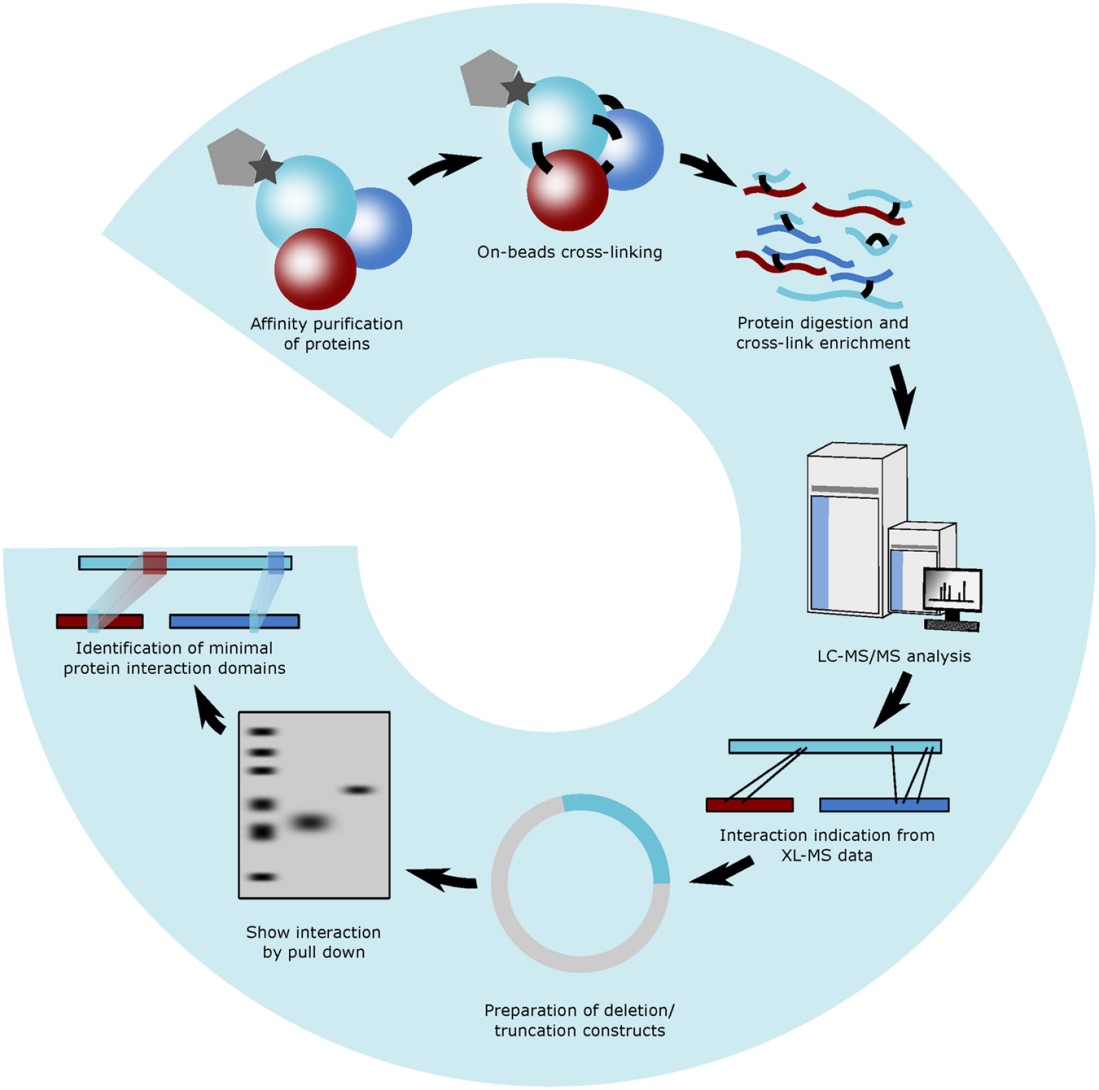
Schematic workflow The schematic workflow presents the integrated approach to characterize minimal protein interaction domains. XL-MS is applied to affinity purified protein complexes to identify cross-linked residue pairs. Protein segments were designed based on cross-linking results and the minimal binding domain is further refined by biochemical analysis.

In this study, we sought to explore the capability of XL-MS to direct the characterization of protein interaction interfaces. It has been well established that XL-MS captures amino acid residues that are in close proximity^19^, however, the relationship between cross-linked peptides and their contributions to protein binding has not yet been assessed thoroughly. Here, we focused on two protein complexes of various nature, namely, a complex formed by MICAL3, ELKS and Rab8A, involved in exocytotic vesicle trafficking^20^, and a complex of CLASP2, SLAIN2, and ch-TOG, important for the regulation of microtubule plus end dynamics^21^. The two complexes are functionally related: CLASP2 with associated proteins stabilizes microtubule plus ends in the vicinity of the cell cortex near the sites enriched in ELKS and its partners such as LL5β^22^. ELKS together with MICAL3 and the two Rabs controls the fusion of exocytotic carriers with the plasma membrane^20,23^. Together these protein complexes thus participate in the spatial coordination of microtubule organization, secretory vesicle delivery along microtubules to their plus ends and vesicle fusion. Such coordination is important, for example, for regulating the dynamics of focal adhesions at the leading cell edge^24^. Protein complexes were purified from cell lysates using the biotin (Bio) tag^25^ and thereafter subjected to XL-MS experiments to determine amino acid residues that are in close proximity. These residue-to-residue connectivities were used to guide the design of protein truncations and deletion mutants, thereby confirming minimal binding domains by affinity purification-based assays (Fig. 1).

These experiments allowed us to successfully identify or refine several protein interaction domains. In some examples, the biochemically validated protein interaction domains were in very good agreement with XL-MS results, however in other cases, cross-linking sites were present in a larger sequence region compared to the minimal protein interaction segments. Based on the analysis, we conclude XL-MS provides useful information for the assessment of protein binding domains and has a good potential to be implemented as a standard procedure in the studies of protein interactions. However other approaches, such as affinity purification assays and secondary structure predictions are necessary to complement XL-MS in order to accurately locate minimal domains responsible for specific PPIs.

## Results

### Characterization of the minimal interaction domains of MICAL3, ELKS and Rab8A

The first protein complex we investigated is formed by MICAL3, ELKS and Rab8A. The complex functions in constitutive exocytosis and is required for vesicle docking and fusion with the plasma membrane^20^. It has been shown previously that Rab8A and ELKS are linked by MICAL3, however, the molecular details of their interactions were not elucidated^20^. To characterize their binding sites, we used cells overexpressing either BioGFP-MICAL3 and BFP-Rab8A or BioGFP-MICAL3, BFP-Rab8A, mCherry-ELKS and mCherry-Rab6. In both cases, we isolated protein complexes by single-step streptavidin-based purification using BioGFP-MICAL3 as the bait and cross-linked them on-beads using disuccinimidyl sulfoxide (DSSO) cross-linker. Subsequently, the proteins were enzymatically digested, and the cross-linked peptides were analysed by MS.

**Figure 2:**
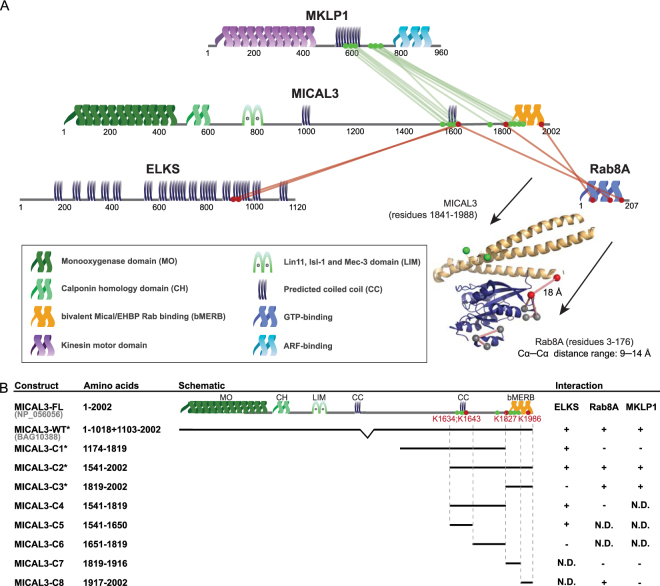
Cross-link mapping and characterization of binding domains in the complex formed by MICAL3, ELKS and Rab8A. (**A**) Cross-link mapping of interactions between MICAL3, ELKS and Rab8A. Cross-linking data of the MICAL3-MKLP1 complex from reference^33^ are included for comparison. Protein domains are depicted based on Uniprot database: MO (monooxygenase domain), CH (calponin homology domain), LIM (Lin11, Isl-1 and Mec-3 domain (zinc binding), CC (predicted coiled coil) and bMERB (bivalent Mical/EHBP Rab binding domain). Inter-protein cross-links between MICAL3, ELKS and Rab8A are shown as red lines and corresponding lysine residues are labelled as red dots. Inter-protein cross-links in the MICAL3-MKLP1 complex are shown as green lines and corresponding lysine residues are labelled as green dots. Cross-link mapping of Rab8A-MICAL3 is based on the structure of the complex formed by Rab8A and the C-terminal part of MICAL-CL (PDB: 5SZI). Protein 3D structures are generated using Pymol v1.5. Lysine residues are depicted in colours based on their origins: red: Lys from MICAL3-Rab8A inter-links; green: Lys from MICAL3-MKLP1 inter-links; grey: Lys from Rab8A intra-protein cross-links. (**B**) An overview of MICAL3 truncation constructs and their interactions with ELKS, Rab8A or MKLP1. *:published information from reference^20^ or reference^33^.

XL-MS analysis revealed 93 cross-links, including 57, 24, 7 intra-protein cross-links on MICAL3, ELKS and Rab8A respectively, as well as 2 inter-protein cross-links between MICAL3 and ELKS, and 3 cross-links between MICAL3 and Rab8A (Fig. 2A, Supplementary Figure S1A–C, Supplementary Table). No cross-links were found on Rab6, although Rab6A is known to directly interact with ELKS^26^. To analyse our cross-linking data, we first compared intra-protein cross-links to the available high-resolution structures of proteins and protein domains. MICAL3 consists of 2002 amino acids and contains an N-terminal monooxygenase (MO) domain, followed by a calponin homology (CH) domain, a Lin11, Isl-1 and Mec-3 (LIM) domain and a C-terminal bivalent MICAL/EHBP Rab binding (bMERB) domain^20,27^. Out of 57 intra-protein cross-links in MICAL3, nine cross-links could be mapped onto a structure homologue of the N-terminal MO domain (PDB: 4TXK, a crystal structure of the MO and CH domain-containing fragment of MICAL1) and two cross-links were located within the structure available region of the C-terminal bMERB domain (PDB: 5SZG). While nine cross-links fall below 30 Å (~23.4 Å + 5 Å tolerance for protein flexibility in solution), which is the expected distance range of DSSO, the two cross-links connecting the MO and CH domain of MICAL3 clearly violate the maximal distance restraint (Supplementary Figure S1A). Since the CH domain is connected to the MO domain by a flexible linker^28^, these two inter-domain cross-links likely indicate a different relative positioning of these two domains in solution^28^. In addition, we also examined the intra-protein cross-links of Rab8A and ELKS. For Rab8A, six out of seven cross-links could be mapped and fully complied with the high-resolution structure (PDB: 5SZI, a crystal structure of the Rab8A and C-terminal part of MICAL-CL) (Fig. 2A, Supplementary Figure S1C). Since no structure is available for ELKS, intra-protein crosslinks in this protein could not be evaluated in this manner.

We also identified multiple cross-links connecting the N- and C-terminal parts of MICAL3, in line with the notion that MICAL1 and MICAL3 can be autoinhibited through inter-domain interactions^29,30^

Interestingly, a cross-linking pattern completely different from that of MICAL3 was observed for ELKS. The observation that cross-links are extremely close in sequence strongly indicate ELKS forms an elongated parallel coiled coil, which is in line with the previous analyses of this protein^31,32^ (Supplementary Figure S1B).

In addition to intra-protein cross-links, three inter-protein cross-links between MICAL3 and Rab8A and two inter-protein cross-links between MICAL3 and ELKS were observed (Fig. 2A, Supplementary Table), corresponding to four lysines of MICAL3, two lysines of ELKS and three lysines of Rab8A. One out of three cross-links between Rab8A and MICAL3 could be mapped on the structure and was in agreement with the high-resolution structure of human Rab8A in complex with the bMERB domain of MICAL-CL, the homologue of the C-terminal part of MICAL3 (PDB: 5SZI) (Fig. 2A).

**Figure 3:**
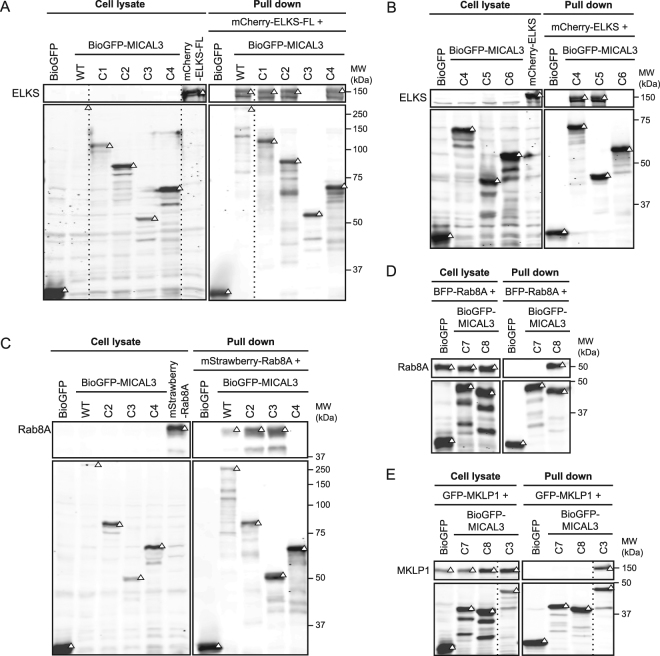
Characterization of the domains responsible for the interactions between MICAL3, ELKS and Rab8A. Results of streptavidin pull down assays. In all panels, triangles indicate signals at the expected molecular weight. Dashed lines indicate cropped lanes irrelevant for this study. Full images are presented in Supplementary Figure S3. (**A**,**B**) Pull downs are performed by mixing the lysates of cells co-expressing biotin ligase BirA and differently truncated BioGFP-tagged MICAL3, with the lysates of cells expressing mCherry-ELKS-FL. Rabbit-anti-ELKS antibody and mouse-anti-GFP antibody were used for western blotting, depicted in the upper and lower boxes respectively. (**C**) Illustrated pull down is performed by mixing the lysates of cells co-expressing biotin ligase BirA and differently truncated BioGFP-tagged MICAL3, with the lysates of cells expressing mStrawberry-Rab8A. The blot is incubated with rabbit-anti-Rab8A and mouse-anti-GFP antibodies for the upper and lower boxes. (**D**) The pull down is performed by mixing the lysates of cells co-expressing biotin ligase BirA and indicated BioGFP-tagged MICAL3 constructs with lysates of cells expressing BFP-Rab8A. Western blot is performed using rabbit-anti-Rab8A and mouse-anti-GFP antibodies for the upper and lower boxes. (**E**) Pull down is performed by the mixing of lysates of cells co-expressing biotin ligase BirA with indicated BioGFP-tagged proteins with lysates of cells expressing GFP-MKLP1. Mouse-anti-GFP antibody is used in both upper and lower boxes.

To assess the binding efficacy of cross-linked regions, we designed eight truncation mutants of MICAL3, each of which includes none, one, two, three or all four lysine residues that are cross-linked with either ELKS or Rab8A (Fig. 2B). For MICAL3 and ELKS interaction, four out of six constructs of MICAL3 (MICAL3-C1, C2, C4 and C5) show binding affinity to ELKS. The shortest construct sufficient for the interaction, MICAL3-C5, encompassed a single coiled coil region and the two lysine residues involved in the MICAL3-ELKS cross-linking (Figs 2B and 3A,B). Similarly, five truncation constructs of MICAL3 were also used for pull-down assays to investigate the interactions with Rab8A protein (Figs 2B and 3C,D). Three truncation constructs, MICAL3-C2, C3 and C8, were found to retain the binding affinity of Rab8A, demonstrating that the minimal Rab8A-interaction domain of MICAL3 locates at the C-terminal half of the bMERB region (MICAL3-C8), encompassing a single cross-linked residue. These results are in agreement with the cross-linked lysine residues identified in our XL-MS experiment, but also show that the cross-linked regions can be broader than the minimal interaction domains.

While bMERB engages in Rab8A binding, it is also responsible for the interaction with MKLP1, as we have demonstrated in a previous study^33^. In our previous work we found that ten lysines of MICAL3 were cross-linked with MKLP1^33^. Two of these lysines (K1634 and K1827) were also cross-linked with Rab8A (Fig. 2A), however, they were not within the minimal binding region (MICAL3-C8) responsible for the binding to Rab8A. Among the other MICAL3 lysines that were cross-linked to MKLP1, two could be mapped to the known bMERB structure. Interestingly, they both localize to the bMERB surface which, based on the structure of the Rab8A-MICAL-CL complex, is not directly involved in the interaction with Rab8A (Fig. 2A). This might suggest that the binding sites of MICAL3 to MKLP1 and Rab8A are distinct. To test this hypothesis, we investigated the interaction between MKLP1 and the two fragments of the MICAL3 bMERB domain (MICAL3-C7 and C8) and found that neither MICAL3-C7 nor C8 were sufficient for binding to MKLP1 (Fig. 3E). These results show that the binding mode of Rab8A and MKLP1 to MICAL3 is indeed different: the C-terminal part of bMERB is sufficient for Rab8A binding, while a larger region of MICAL3 C-terminus is needed for MKLP1 interaction. Presence of distinct cross-links within MICAL3-Rab8A and MICAL3-MKLP1 complexes is in agreement with this result.

**Figure 4:**
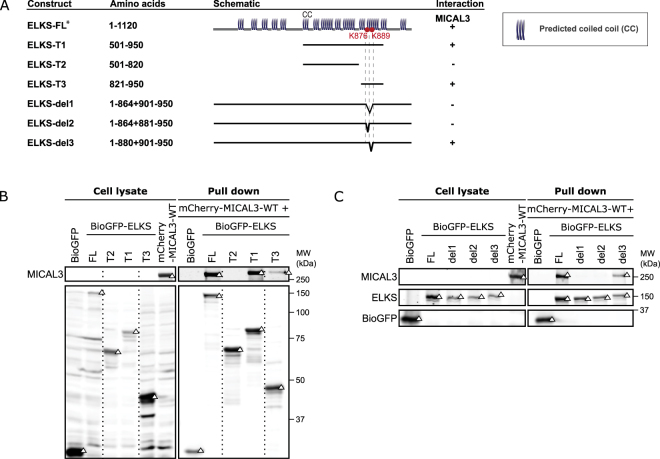
Mapping of the MICAL3-interacting site in ELKS. (**A**) An overview of ELKS truncation and deletions constructs and their interactions with MICAL3. (**B**,**C**) Results of streptavidin pull down assays, performed by mixing the lysates of cells co-expressing biotin ligase BirA and differently truncated or deleted BioGFP-tagged ELKS, with the lysates of cells expressing mCherry-MICAL3-WT. Rabbit-anti-MICAL3 and mouse-anti-GFP antibodies were used for the upper and lower boxes. Triangles indicate signals at the expected molecular weight. Dashed lines indicate cropped lanes irrelevant for this study. Full images are presented in Supplementary Figure S4. *Published information from reference^20^.

Next, we generated a set of truncation and deletion constructs of ELKS to better define the MICAL3-ELKS interaction (Fig. 4A). Two C-terminal domain fragments (ELKS-T1, and T3) were found positive for binding to the full-length MICAL3, while no binding was observed for ELKS-T2 (Fig. 4A,B). These data suggest ELKS-T3 construct, which comprises both cross-linked lysine residues of ELKS, is sufficient for MICAL3-ELKS interaction. Moreover, to further assess the roles of the two cross-linked lysine residues (K876 and K889) of ELKS, we designed three deletion mutants (ELKS-del1, del2 and del3) for affinity purification assays (Fig. 4A–C). Remarkably, the region adjacent to K876 (corresponding to ELKS-del2), but not the region adjacent to K889 (corresponding to ELKS-del3) contained amino acid sequences that are essential for the interaction. In this example, small deletions in ELKS were designed directly based on XL-MS results, allowing accurate and rapid localization of the exact sites essential for MICAL3-ELKS interaction.

### Characterization of the minimal interaction domains of SLAIN2, CLASP2 and ch-TOG

**Figure 5:**
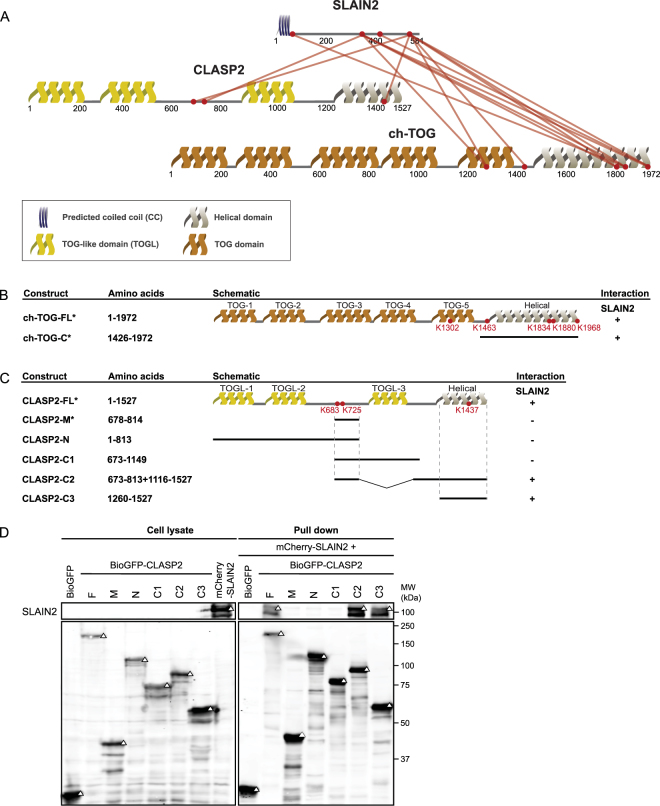
Cross-link mapping and characterization of the domains responsible for the interactions between SLAIN2, CLASP2-and ch-TOG. (**A**) Cross-link mapping on the complex formed by SLAIN2, CLASP2 and ch-TOG. Protein domains are indicated as CC (predicted coiled coil), TOG (tumour overexpressed gene) and TOG-L (TOG-like) domains. (**B**) Ch-TOG truncation constructs and their interaction with SLAIN2. (**C**) An overview of CLASP2 truncation constructs and their interactions with SLAIN2. (**D**) Results of streptavidin pull down assay, performed by mixing of lysates of cells co-expressing biotin ligase BirA and indicated BioGFP-tagged CLASP2, with the lysates of cells expressing mCherry-SLAIN2. Mouse-anti-mCherry and rabbit-anti-GFP antibodies were used for the upper and lower boxes respectively. Triangles indicate signals at the expected molecular weight. Full western blot image is presented in Supplementary Figure S5. *Published information from reference^21^.

The second protein complex we examined is formed by SLAIN2, CLASP2 and ch-TOG, known to be important for the regulation of microtubule dynamics^21^. It has been  shown that SLAIN2 weakly binds to CLASP2 and interacts with ch-TOG with a much higher affinity^21^. To further investigate the interaction details of this complex, we performed XL-MS experiments with purified SLAIN2-CLASP2-ch-TOG complex from the extracts of cells overexpressing BioGFP-SLAIN2 and GFP-CLASP2 using BioGFP-SLAIN2 as the bait. Ch-TOG was not overexpressed in this assay, and thus the detected ch-TOG peptides were derived from endogenous protein. We identified 69 cross-links, including 49 intra-protein cross-links within SLAIN2, 3 intra-protein cross-links within ch-TOG, 3 intra-protein cross-links within CLASP2, 3 inter-protein cross-links between SLAIN2 and CLASP2 and 11 inter-protein cross-links between SLAIN2 and ch-TOG (Fig. 5A, Supplementary Figure S2, and Supplementary Table).

In SLAIN2-CLASP2-ch-TOG complex, none of the intra-protein cross-links are within the regions of known structure. As high-resolution structure of TOG domain is available, we therefore generated homology models for TOG-5 (TOG-5_1168–1422_) and TOG-helical (TOG-helical_1505–1970_) domains using Phyre2^34^ and mapped the three intra-protein cross-links within these two regions onto the homology models (Supplementary Figure S2A). All three cross-links are in agreement with the maximal distance restraint provided by the DSSO cross-linker.

Next, we move to the analysis of the inter-protein cross-links. Of the 11 inter-protein cross-links between SLAIN2 and ch-TOG, all cross-linked lysine residues of ch-TOG are located within the C-terminal part, in line with our previous reported binding domain of ch-TOG with SLAIN2 (TOG-C1_1426–1972_, Figure 5A,B)^21^. Similar to the results obtained from MICAL3 and ELKS, one of the four cross-linked lysine residues in ch-TOG, K1302 located in TOG-5, is outside of previously mapped domain essential for the interaction with SLAIN2^21^.

The cross-linked lysine residues of SLAIN2, however, span the entire sequence of this protein (Fig. 5A), which is contradictory to the minimal region responsible for binding of SLAIN2 to chTOG (SLAIN2_1–267_) defined previously^21^. We reason that the dispersed cross-linking pattern may likely due to the intrinsic disorder of SLAIN2, the sequence of which is rich in basic residues and has been predicted previously as largely unstructured^21^. The cross-linking results of SLAIN2 indicate XL-MS is prone to present cross-links that are unrelated to direct binding for intrinsically disordered proteins.

We designed five truncation constructs of CLASP2, covering two or three cross-linked lysine residues (CLASP2-M, CLASP2-N, CLASP2-C1 and CLASP2-C2 and CLASP-C3) and performed affinity purification to characterize the interaction domains of SLAIN2 and CLASP2 (Fig. 5C,D). We observed that two constructs, CLASP2-C2 and CLASP-C3, retain the binding affinity to SLAIN2, indicating that only the C-terminal region adjacent to K1437, which is predicted to be helical, is essential for the binding to SLAIN2 (Fig. 5A,C–D). In contrast, the intrinsically disordered region in the middle of CLASP2 did not bind to SLAIN2, in spite of showing two cross-links with this protein. Cross-linking results of SLAIN2 and CLASP2 thus also encompass a larger region compared to the minimal binding domain determined by biochemical assays, and the disordered protein regions in these proteins appear to be more accessible in cross-link formation.

## Discussion

XL-MS, which has been widely applied to facilitate structural modelling of proteins/complexes and docking of interaction interfaces^15,16,35–37^, has also been used to direct mutagenesis to disrupt the surface interactions in several cases^38–40^. However, the question of how accurate cross-linked residues and neighbouring regions reflect the minimal binding site of the interaction has not been investigated in detail. Here, we examined this issue using XL-MS in combination with deletion analysis in two illustrative examples. In the complex formed of MICAL3, Rab8A and ELKS, cross-linked sites fitted reasonably well with the interaction interfaces determined by biochemical assays, whereas in the analysis of the interaction interfaces between SLAIN2, CLASP2 and ch-TOG, the match between the protein fragments that are required for protein binding and the cross-linking results was less good. Despite some discrepancy, cross-linking sites provided valuable guidance for designing truncation and deletion mutants to narrow the interaction domains. However, the exact sites of interaction needed to be further validated by biochemical methods, as cross-links may likely be boarder than the minimal protein interaction regions.

A nice example of XL-MS guided mapping is provided by identifying a short amino acid stretch of ELKS (residues 865–880) essential for the interaction with MICAL3, and the identification of the minimal ELKS-interacting domain in MICAL3. Similarly, in our recent study describing the interaction between MICAL3 and the centralspindlin component MKLP1, XL-MS data also helped us to rapidly generate small deletions in MKLP1 to disrupt its binding to MICAL3, allowing functional assessment of the interaction^33^.

In addition to evaluating the power of XL-MS, our study allowed refining protein interaction domains in the two investigated protein complexes. For example, we compared lysine residues of MICAL3 that were cross-linked with either MKLP1 or Rab8A. A recent publication characterized the structure of the bMERB domain of MICAL family in complex with Rab8 family proteins and found two adjacent Rab binding sites located in the N- and C-terminal portion of the bMERB domain of MICAL1^27^. Interestingly, our mapping showed that Rab8A interacts with MICAL3 through the C-terminal half of the bMERB domain of MICAL3 (MICAL3-C8), while the entire bMERB domain (MICAL3-C3) of MICAL3 was needed for binding to MKLP1, in this was reflected in the distinct cross-linking patterns in these protein complexes (Fig. 2A). These data might point to the ability of the bMERB domain of MICAL3 to interact with MKLP1 and Rab8A simultaneously. Such an interaction mode would be in agreement with our previously proposed model, in which MICAL3 acts as a midbody-associated protein hub that can bring together MKLP1, ELKS and Rab8A and in this way promote maturation of the intercellular bridge and abscission^33^. The fact that the minimal ELKS-binding domain of MICAL3 does not overlap with the bMERB domain needed for binding to Rab8A or MKLP1 supports this idea.

In the other example analysed here, we showed that SLAIN2 binds to the C-terminal helical domains of CLASP2, similar to most of the other CLASP binding partners, such as CLIP-170 and CLIP-115^41^, LL5β^22^, GCC185^42^, the kinetochore motor centromere protein E (CENP-E), and the chromokinesin Xkid^43^, indicating that CLASP likely engages with its partners in independent protein complexes to stabilise microtubules in different contexts. These data will be useful for dissecting the detailed mechanisms underlying the functions of the studied complexes.

In summary, we show XL-MS facilitates the characterization of minimal protein interaction domains. XL-MS studies can be performed under physiological conditions with modest amounts of material, and in some cases, as exemplified by identification of cross-links between SLAIN2 and ch-TOG, applicable to proteins at endogenous expression levels. This is clearly a major advantage in comparison to methods that require highly pure and large amount of proteins. Another advantage of XL-MS, comparing to computational means for binding site prediction, is that XL-MS does not require any prior structural information of proteins or protein homologues, which is an essential element for meaningful predictions in many circumstances. However, the predictive power of XL-MS depends on the structural properties of proteins: for the protein complexes analysed here, the method clearly performed much better with folded entities than intrinsically unstructured polypeptides. Taken together, with the knowledge of the advantages and limitations of the methodology, XL-MS is likely to become an important addition to the routine methods of PPI characterization.

## Materials and Methods

### DNA constructs

The following constructs were used in the study: protein-biotin ligase BirA, BioGFP, BFP-Rab8A, mCherry-Rab6, BioGFP- and mCherry-ELKS, BioGFP- and mCherry-MICAL3-WT, BioGFP-SLAIN2 and GFP-CLASP2^20–22^, as well as GFP-MKLP1^33^. MICAL3 truncation mutants were derived from BioGFP-MICAL3 using a PCR-based strategy and subcloned into mCherry- and BioGFP-expressing vectors. ELKS truncation and deletion mutants were obtained from GFP-ELKS using a PCR-based strategy in combination with Gibson assembly.

### Antibodies

The following primary antibodies were obtained from commercial sources: mouse-anti-mCherry (Clontech), rabbit-anti-GFP (Abcam, ab290), mouse-anti-GFP (Roche, 11814460001), rabbit-anti-Rab8 (D22D8) (Cell Signaling Technology, #697), and rabbit anti-ELKS (Proteintech, 22211-1-AP). Polyclonal rabbit-anti-MICAL3 antibody were custom raised and has been used previously^20^. Secondary antibodies were purchased from commercial sources: IRDye 800CW/680LT goat anti-rabbit, and goat anti-mouse (Li-Cor Biosciences).

### Cell culture and pull down assays

HEK293T cells were purchased from ATCC and passaged in 1:1 mixture of DMEM and Ham’s F10 medium (Lonza) supplemented with 10% fetal calf serum and 1% Penicillin-Streptomycin solution (Sigma). Cell lines were routinely checked for mycoplasma contamination using LT07-518 Mycoalert assay (Lonza). HEK293T cells were transfected with either a BioGFP-fusion construct (pull down bait) together with a plasmid encoding the biotin ligase BirA or an mCherry-, mStrawberry-, GFP- or BFP- fusion construct (pull down prey) for 24 hr using Polyethylenimine (Polysciences). Transfected cells were lysed in the lysis buffer (100 mM Tris-HCl pH 7.5, 150 mM NaCl, 1% Triton X-100, supplemented with protease inhibitors (Roche)) and cell lysates were cleared by centrifugation at 13,200 rpm for 20 minutes. Dynabeads® M-280 streptavidin (Invitrogen) were pre-treated in blocking buffer (50 mM Tris-HCl pH 7.5, 150 mM NaCl, 1% Triton X-100, and 0.02% chicken egg albumin) for 1 hour at 4 °C prior to pull down assays. For pull down assays, lysates of cells expressing a BioGFP- and of cell expressing the prey protein, were mixed and incubated with the beads at 4 °C for 2 hours to allow formation of the protein complexes. Beads were washed four times in ice-cold washing buffer (50 mM Tris-HCl pH 7.5, 150 mM NaCl, 0.5% Triton X-100) using a magnet (Invitrogen), the proteins were eluted by adding 2 × SDS-PAGE sample buffer and heating at 95 °C for 5 min. For successive Western Blot analysis, the samples were equally loaded onto either 8% or 10% SDS-PAGE gels, and run on 120 V in a Mini-PROTEAN® cassette (Bio-Rad). The samples were subsequently transferred to nitrocellulose membranes using a semi-dry blotting setup (Bio-Rad) and Bjerrum Schafer-Nielsen transfer buffer (48 mM Tris, 39 mM glycine, 20% methanol). Blots were blocked with 2% Bovine Serum Albumin in PBS/0.05% Tween-20 for 1 hour at room temperature and incubated with primary antibodies at 4 °C overnight. Blots were washed three times with 0.05% Tween 20 in PBS for 5 minutes at room temperature and incubated with secondary IRDye 680LT and IRDye 800CW antibodies (LI-COR Biosciences) for 1 hour at room temperature. Subsequently, blots were washed three times before imaging on an Odyssey Infrared Imaging system (LI-COR Biosciences). For cross-linking experiments, HEK293T cells were co-transfected with a BioGFP-fusion construct together with a plasmid encoding BirA and prey construct/constructs for 24 hr using Polyethylenimine (Polysciences). Transfected cells were lysed as described above and the extracts were incubated with pre-treated Dynabeads® M-280 streptavidin (Invitrogen) at 4 °C for 2 hours. Beads were washed four times in ice-cold washing buffer.

### XL-MS sample preparation

Protein/complexes from streptavidin pull-downs were further washed two times in cross-linking buffer (20 mM HEPES, pH 7.8 and 150 mM NaCl) and cross-linked on beads using 1 mM DSSO cross-linker^44,45^. The cross-linking reaction was carried out for 45 min at room temperature and quenched with 20 mM Tris-HCl (pH 7.8) for 30 min at room temperature. Subsequently, the on-beads cross-linked proteins were denatured with 2 mM urea, reduced with 4 mM dithiothreitol at 56 °C for 30 min and alkylated with 8 mM iodoacetamide at room temperature for 30 min in the dark. Proteins were digested using trypsin at an enzyme-to-protein ratio of 1:20 (w/w) at 37 °C for 2 hr. The supernatant was removed from the beads and further digested at 37 °C overnight. Protein digests were desalted using Sep-Pak C18 cartridges (Waters), dried and stored at −20 °C for further use. The desalted digests were fractionated by strong cation exchange chromatography (SCX) using an Agilent 1200 system (Agilent Technologies). Zorbax BioSCX-Series II column (0.8 mm inner diameter, 50 mm length, 3.5 μm) was used. SCX fractions were collected, dried and later SCX fractions which predominantly contain + 4 and higher charged species were subjected to LC/MS analysis.

### LC/MS/MS analysis

Peptides and cross-linked peptides were analysed using an ultra HPLC Agilent 1200 system (Agilent Technologies) coupled on-line to an Orbitrap Fusion mass spectrometer (Thermo Fisher Scientific). Reversed-phase separation was performed using a 50 cm analytical column (in-house packed with Poroshell 120 EC-C18, 2.7 µm, Agilent Technologies) with a 90 min gradient. Samples were analysed using either CID-ETD-MS2^45^ or CID-MS2-MS3-ETD-MS2 acquisition strategies^46^. In both strategies, peptides of charge states 4–10 were selected for MS2 acquisitions. MS scan range was set to 375–1575 m/z and resolution was set to 60,000. MS2 scans were performed with a resolution of 30,000 and an AGC target of 5 × 10^4^. The precursor isolation width was 1.6 m/z and the maximum injection time was 100 ms. The CID MS2 normalized collision energy was set to 25%. Calibrated charge dependent ETD parameters were enabled. MS3 scans were performed in the ion trap with CID collision energy of 35%; AGC target of 2 × 10^4^ and MS2 isolation window of 3 m/z. The maximum injection time was set to 100 ms.

### Data analysis

Raw data files were converted to *.mgf files using Proteome Discoverer 1.4 software (Thermo Fisher Scientific) with deconvolution option provided by add-on node MS2-Spectrum Processor (IMP, Vienna). XlinkX v2.0 algorithm was used for cross-link identification. The following settings were used: MS ion mass tolerance: 10 ppm; MS2 ion mass tolerance: 20 ppm; MS3 ion mass tolerance, 0.6 Da; fixed modification: Cys carbamidomethyl; variable modification: Met oxidation; allowed number of mis-cleavage: 3. Database comprises the target sequences of each protein assembly concatenated with the same number of decoy sequences. FDR is calculated based on a target-decoy strategy^47^ and 1% FDR was used to report cross-link results. Furthermore, the search was also performed using the full sequences of the proteins that include the tags. Additional cross-links are presented in Supplementary Table and Supplementary Figure S6.

## Electronic supplementary material

Supplementary information

Dataset 1
