# Supplementary material for: Facilitating identification of minimal protein binding domains by cross-linking mass spectrometry
Source: Sci Rep. 2017 Oct 18;7:13453. doi: 10.1038/s41598-017-13663-y (PMC5647383; doi:10.1038/s41598-017-13663-y)
Supplement: Supplementary file 1 — Supplementary information [file 41598_2017_13663_MOESM1_ESM.pdf]

Facilitating identification of minimal protein binding domains by cross-linking mass spectrometry

**Qingyang Liu [1], Sanne Remmelzwaal [1], Albert J. R. Heck [2], Anna Akhmanova [1*]
and Fan Liu [2,3*]**

[1] Cell Biology, Faculty of Science, Utrecht University, Padualaan 8, 3584 CH Utrecht, the Netherlands

[2] Biomolecular Mass Spectrometry and Proteomics, Bijvoet Center for Biomolecular Research and Utrecht Institute for Pharmaceutical Sciences, Utrecht University, 3584 CH Utrecht, the Netherlands

3 Leibniz Institute of Molecular Pharmacology (FMP), Robert-Rössle-Straße 10, 13125 Berlin, Germany

*Correspondence: a.akhmanova@uu.nl (A.A.); F.liu@uu.nl (F.L.)

Supplementary Information

Supplementary Figure S1. Intra-protein cross-link mapping of MICAL3, ELKS and Rab8A proteins.

Protein domains and high-resolution structures of (A) MICAL3 (PDB: 4TXK and PDB: 5SZG), (B) ELKS and (C) Rab8A. Protein schemes are the same as in Fig.2. Cross-links are shown as grey lines. Cross-links that are mapped onto high-resolution structures are shown in salmon.

Supplementary Figure S2. Intra-protein cross-link mapping of SLAIN2, CLASP2 and ch-TOG proteins.

Protein domains of (A) ch-TOG, (B) CLASP2 and (C) SLAIN2. Protein domains are depicted in the same way as in Fig.5. Cross-links are shown as grey lines. Homology models for TOG-5 (TOG-51168-1422) and TOG-helical (TOG-helical1505-1970) domains are generated using Phyre2 (http://www.sbg.bio.ic.ac.uk/~phyre2) with all default parameters. Cross-links that are mapped onto high-resolution structures are shown in salmon.

Supplementary Figure S3: Original western blots of Figure 3.

A-E) Corresponding original western blots of Figure 3. Red boxes illustrate blot area used in Figure 3. *: MICAL3 truncation construct 1054-2002. **: MICAL3 truncation construct 1174-2002. ***: A different plasmid preparation of BioGFP-MICAL3-C3 construct.

Supplementary Figure S4: Original western blots of Figure 4.

A,B) Original western blots from Figure 4B and C, respectively. Red boxes illustrate blot area used in Figure 4B and C. *: ELKS truncation construct of amino acids 1-440. **: ELKS truncation construct 441-950. ***: ELKS truncation construct 608-950. ****: ELKS truncation construct 731-950.

Supplementary Figure S5: Original western blot of Figure 5.

A) Original western blot from Figure 5D. Red boxes illustrate blot area used in Figure 5D.

Supplementary Figure S6: Additional cross-links detected in BioGFP-tagged MICAL3 and BioGFP-tagged SLAIN2.

(A, B). Cross-link mapping on the GFP tag of MICAL3 and SLAIN2. (C) Cross-links identified between GFP tag and SLAIN2.

Figure S1 Liu et al.

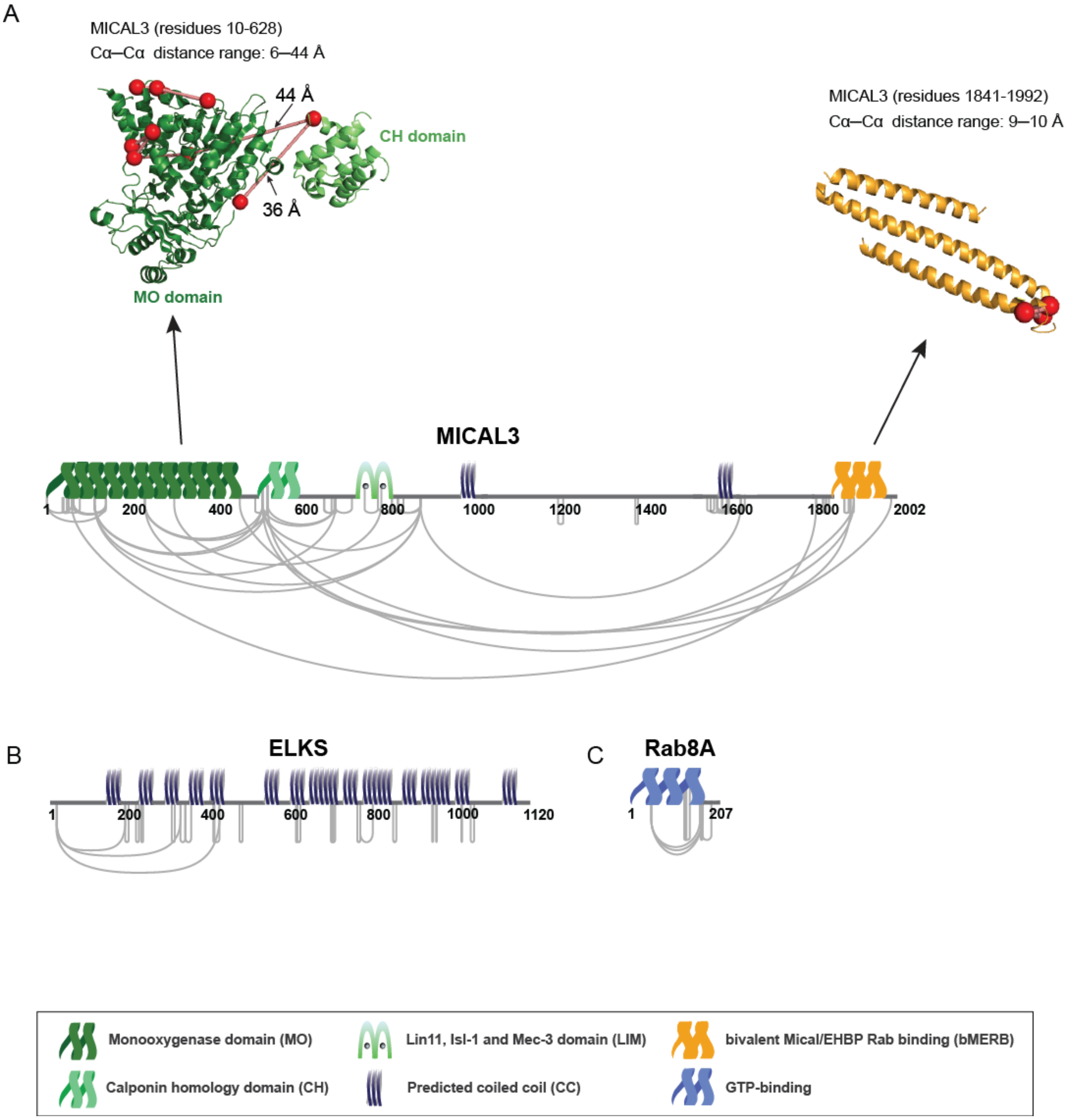

Supplementary Figure S1

Figure S2 Liu et al.

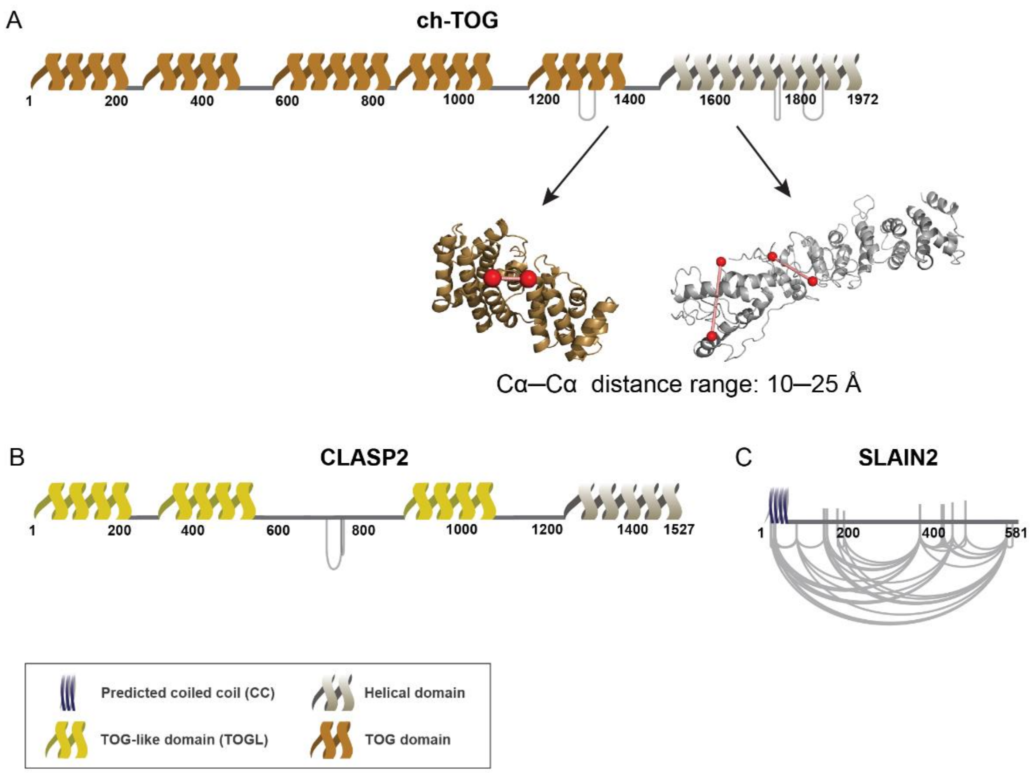

Supplementary Figure S2

Figure S3 Liu et al.

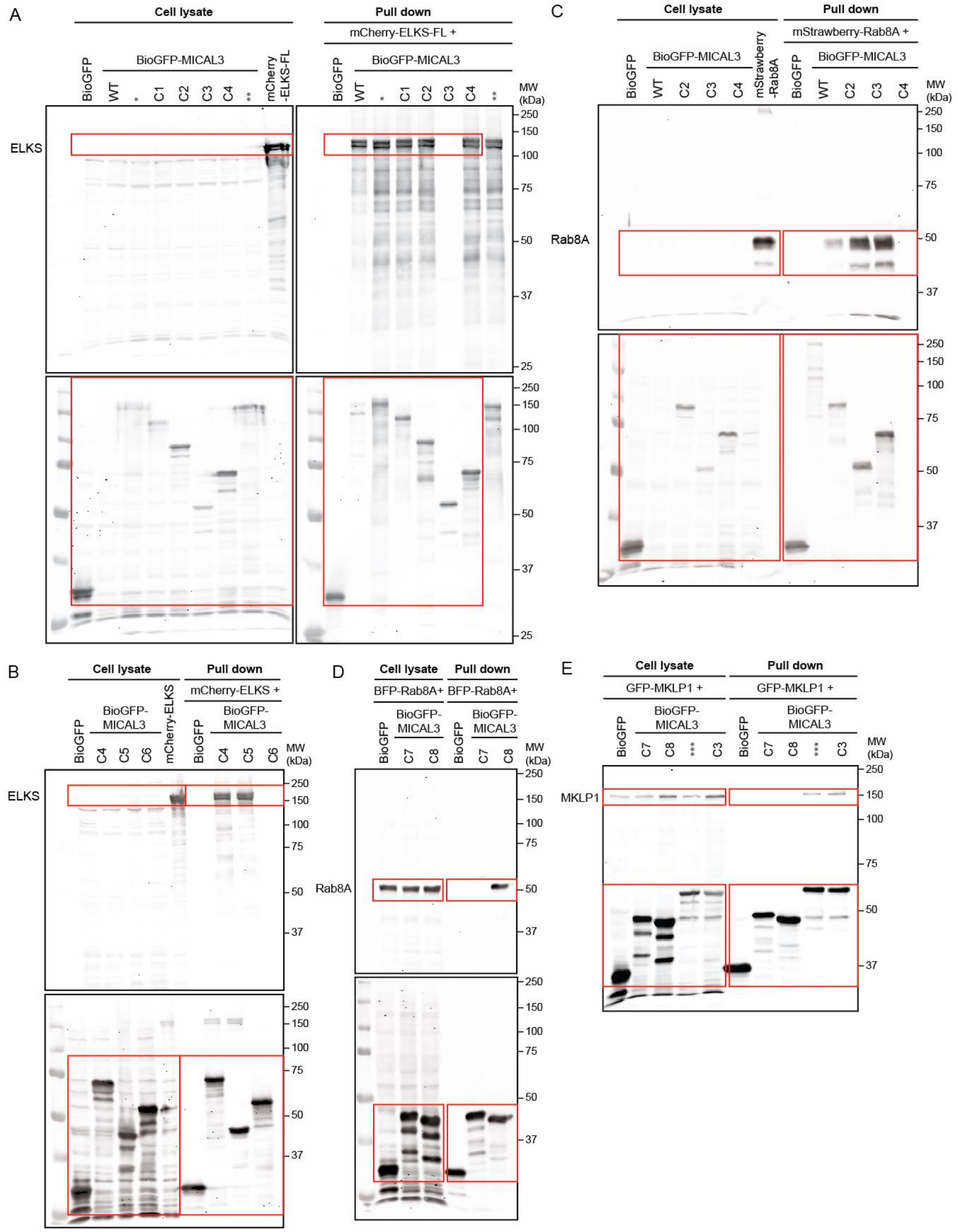

Supplementary Figure S3

Figure S4 Liu et al.

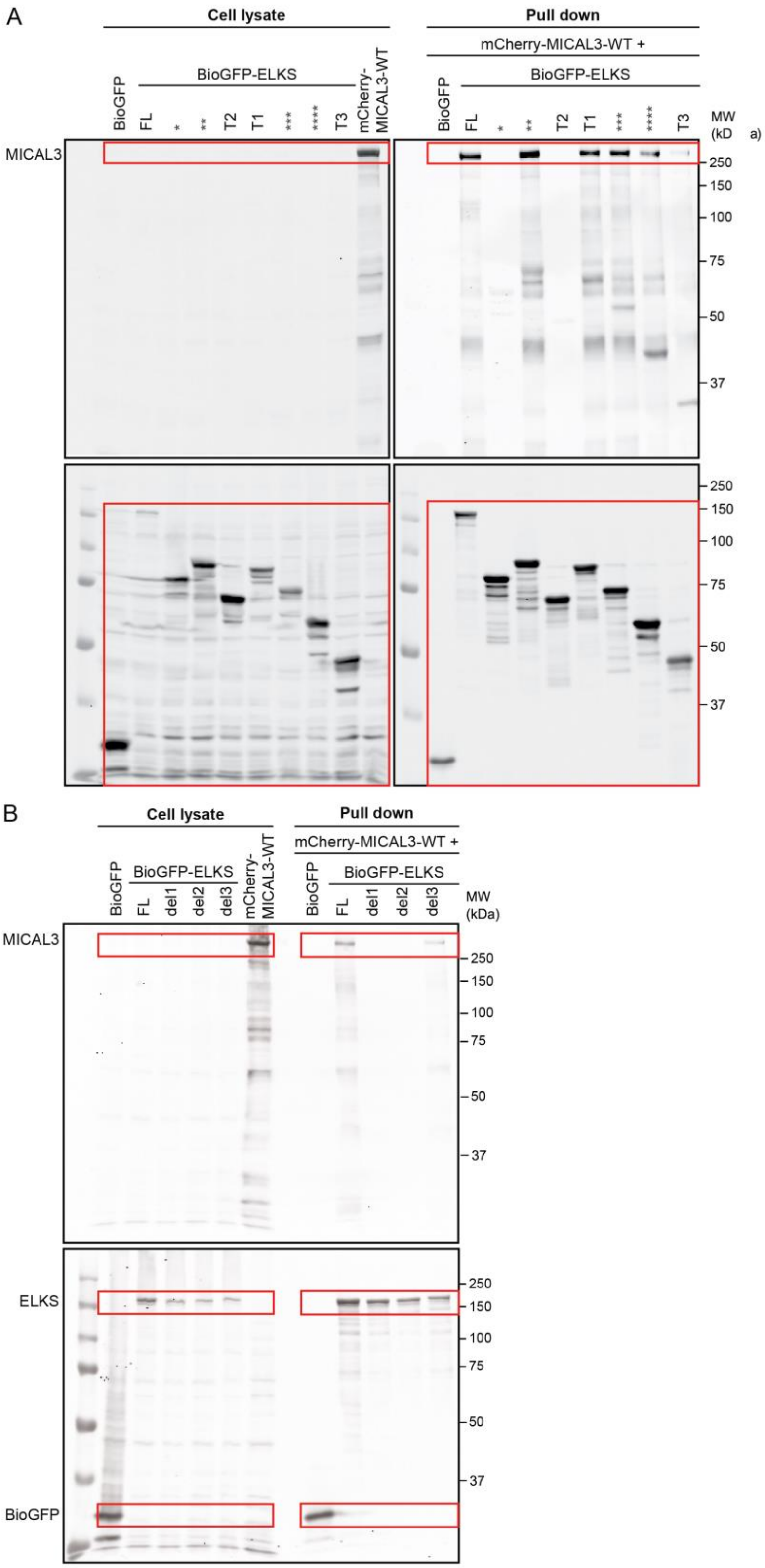

Supplementary Figure S4

Figure S5 **Liu et al.**

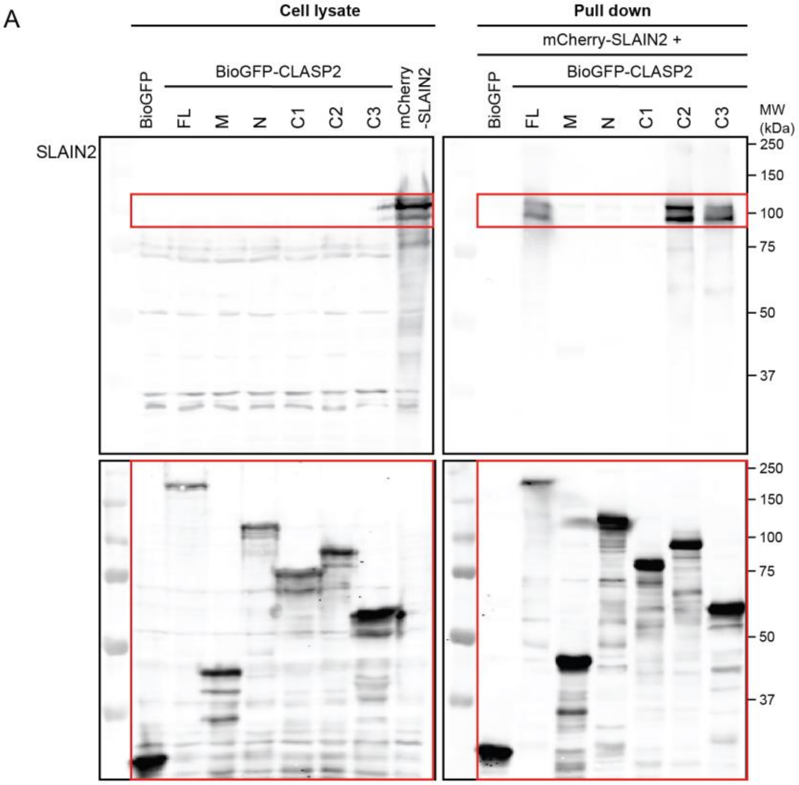

Supplementary Figure S5

Figure S6 **Liu et al.**

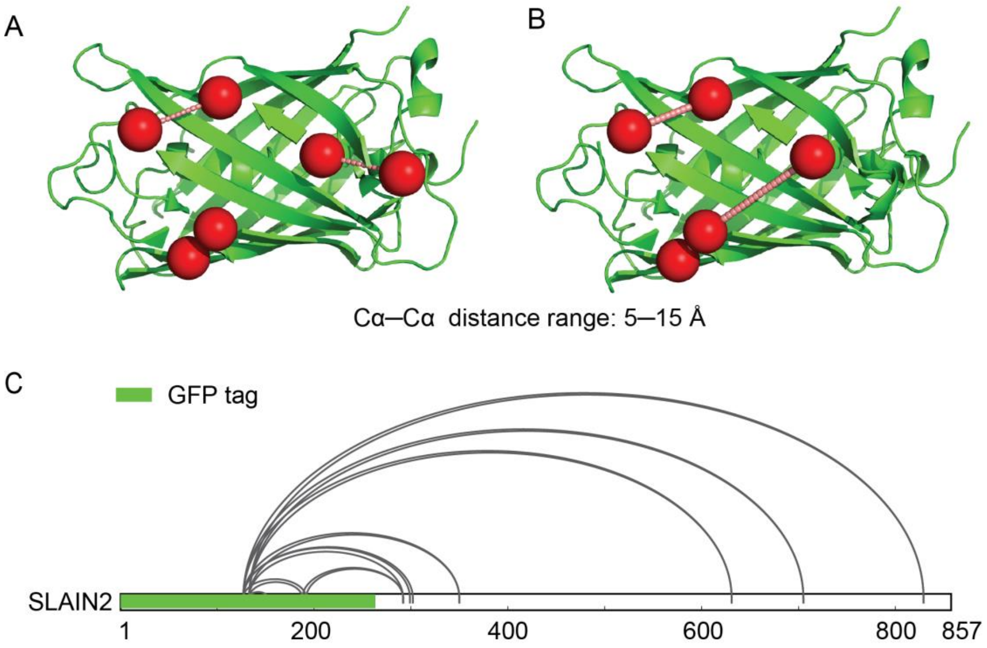

Supplementary Figure S6
